# Sensory-Tactile Functional Mapping and Use-Associated Structural Variation of the Human Female Genital Representation Field

**DOI:** 10.1523/JNEUROSCI.1081-21.2021

**Published:** 2022-02-09

**Authors:** Andrea J. J. Knop, Stephanie Spengler, Carsten Bogler, Carina Forster, Michael Brecht, John-Dylan Haynes, Christine Heim

**Affiliations:** ^1^Charité–Universitätsmedizin Berlin, corporate member of Freie Universität Berlin and Humboldt-Universität zu Berlin, Institute of Medical Psychology, 10117 Berlin, Germany; ^2^Charité–Universitätsmedizin Berlin, corporate member of Freie Universität Berlin and Humboldt-Universität zu Berlin, Berlin Center for Advanced Neuroimaging, 10117 Berlin, Germany; ^3^Bernstein Center for Computational Neuroscience, 10115 Berlin, Germany; ^4^Max Planck Institute for Human Cognitive and Brain Sciences, 04103 Leipzig, Germany; ^5^Department of Biology, Humboldt-Universität zu Berlin, 10117 Berlin, Germany; ^6^Department of Psychology, Humboldt-Universität zu Berlin, 10115 Berlin, Germany; ^7^Department of Biobehavioral Health, The Pennsylvania State University, University Park, PA 16802

**Keywords:** functional mapping, genital field, individual variability, plasticity, sexual behavior, somatosensory cortex

## Abstract

The precise location of the human female genital representation field in the primary somatosensory cortex (S1) is controversial and its capacity for use-associated structural variation as a function of sexual behavior remains unknown. We used a functional magnetic resonance imaging (fMRI)-compatible sensory-tactile stimulation paradigm to functionally map the location of the female genital representation field in 20 adult women. Neural response to tactile stimulation of the clitoral region (vs right hand) identified individually-diverse focal bilateral activations in dorsolateral areas of S1 (BA1–BA3) in alignment with anatomic location. We next used cortical surface analyses to assess structural thickness across the 10 individually most activated vertices per hemisphere for each woman. We show that frequency of sexual intercourse within 12 months is correlated with structural thickness of the individually-mapped left genital field. Our results provide a precise functional localization of the female genital field and provide support for use-associated structural variation of the human genital cortex.

**SIGNIFICANCE STATEMENT** We provide a precise location of the human female genital field in the somatosensory cortex and, for the first time, provide evidence in support of structural variation of the human genital field in association with frequency of genital contact. Our study represents a significant methodological advance by individually mapping genital fields for structural analyses. On a secondary level, our results suggest that any study investigating changes in the human genital field must map the field individually to achieve sufficient precision. Our results pave the way for future research into the plasticity of the human genital cortex as a function of normal or adverse experience as well as changes in pathologic conditions, i.e., sexual dysfunction, sexual deviation, or sexual risk-taking behavior.

## Introduction

The precise location of the female genital representation field in the primary somatosensory cortex (S1) is still a matter of contention (Di Noto et al., 2013; Cazala et al., 2015). Furthermore, the capacity of the human genital representation field for use-associated structural plasticity has never been studied.

In their first presentation of the somatosensory homunculus, Penfield and Rasmussen (1950) placed the male genital field below the foot in the mesial part of S1. This nonsomatotopic location of the genital field was supported by results demonstrating functional activations in the mesial wall of the paracentral lobe in response to electrical stimulation of the dorsal penile nerve in males (Allison et al., 1996; Nakagawa et al., 1998; Mäkelä et al., 2003) and manual-tactile clitoral, vaginal, and cervical self-stimulation in females (Komisaruk et al., 2011). Other studies provided evidence for a somatotopically-ordered representation of the genital field adjacent to the hip and knee areas by demonstrating activations in dorsolateral regions of the postcentral gyrus in response to electrical stimulation of the dorsal clitoral nerve (Michels et al., 2010) or partner-delivered manual stimulation of the clitoris in females (Georgiadis et al., 2006, 2009), as well as sensory-tactile brushing of the penile shaft in males (Kell et al., 2005). These latter results are in line with evidence from rodent studies that localize the rat genital cortex in somatotopic order and bilateral symmetry (Lenschow et al., 2016; Lenschow and Brecht, 2018).

The mode of stimulation used in functional mapping studies may contribute to heterogeneous results concerning the location of the genital field in humans. Specifically, electrical stimulation is not equivalent to sensory touch and elicits less focal responses (Pratt et al., 1980; Forss et al., 1994). Self-delivered or partner-delivered manual stimulation includes touching of areas adjacent to the genitals and elicits sexual arousal that may confound neural response (Georgiadis et al., 2006, 2009, 2010; Komisaruk et al., 2011). The only study using a focal sensory-tactile nonarousing stimulation paradigm in the form of soft brushing of the penile shaft was limited to men and does not inform about female genital field location (Kell et al., 2005). Indeed, no study to date has functionally mapped the female genital field in humans using a magnetic resonance imaging (MRI)-compatible focal sensory-tactile nonarousing stimulation paradigm, contrasting neural response to sensory stimulation of the clitoris against sensory stimulation of a control region.

Commensurate with the fact that the precise location of the genital field remains controversial, there is no evidence regarding its capacity for structural change in association with use in humans. It is well established that the human brain has substantial capacity for plasticity as a function of experience (Draganski and May, 2008). Use-dependent structural reorganization of human S1 has been observed after deprivation of afferent input because of limb amputation (Elbert et al., 1994; Flor et al., 1995; Knecht, 1998) or peripheral nerve lesion (Henderson et al., 2011). Whether or not the human genital field is capable to structurally adapt to its normal use is entirely unknown. Recent evidence suggests that the developing rat genital cortex expands with genital stimulation, facilitating puberty (Lenschow et al., 2017; Sigl-Glöckner et al., 2019).

We here combine the investigation of the location of the female genital field with the question of structural variation of this field as a function of sexual behavior, considering the important issue of individual variability: (1) we provide a precise localization the human female genital representation field by using a focal sensory-tactile nonarousing stimulation paradigm during functional MRI (fMRI) to contrast neural response of stimulation of the clitoral region versus the right hand. (2) We use individually-mapped genital fields based on the 10 most activated vertices per hemisphere for each woman and assess structural thickness in the individually-mapped field using cortical surface analysis. (3) We show that thickness of the individually-mapped genital field varies with the frequency of sexual intercourse in the past 12 months, compatible with use-associated plasticity.

## Materials and Methods

### Sample

We recruited 25 adult healthy women aged 18–45 years. General exclusion criteria applied to select women were lifetime or current psychiatric disorders, exposure to childhood abuse or neglect (including sexual abuse), neurologic disorders, physical disease, central nervous system or urogenital surgery, psychotropic medication within six months, sexually transmitted disease, sexual disorders (including sexual anxiety, discontent or dysfunction or dissociation during sexual activity), past or current pregnancy, and current menstruation. Exclusionary conditions were assessed using clinician-administered interviews and standard questionnaires (Oldfield, 1971; Hahlweg, 1996; McGahuey et al., 2000; Berner et al., 2004; Kühner et al., 2007; Brenk-Franz and Strauß, 2011; Hansen et al., 2012; Klinitzke et al., 2012; Hoyer et al., 2015; Müller, 2016). Women were screened for contraindications of MRI scanning. Of the 25 women recruited into the study, 20 women were included in the analyses. Five women were excluded because the experimental procedure (i.e., genital stimulation paradigm) was not successful.

### Procedure

Women underwent a standardized study visit at the Institute of Medical Psychology and the Berlin Center for Advanced Neuroimaging, both at Charité−Universitätsmedizin Berlin. During the visit, women underwent all study procedures, including interviews and questionnaires for demographics and exclusionary conditions. To localize the genital representation field in S1, women underwent (1) fMRI scanning during sensory-tactile stimulation of the clitoris versus dorsum of the right hand; (2) structural MRI to assess thickness of the individually mapped genital field; and (3) a detailed sexual history to assess frequency of sexual intercourse, i.e., genital sensory touch, in the past year and lifetime for the assessment of use-dependent plasticity of the individually mapped genital field. The study was approved by the institutional ethics committee and was conducted in accordance with the Declaration of Helsinki. Written informed consent of the participants was obtained.

### MRI acquisition

Structural MRI was performed using a 3.0 T Siemens Tim Trio MRI scanner (Siemens Medical System) with a standard 12-channel head coil. Two 1-mm^3^ isotropic T1 anatomic scans were acquired in the sagittal plane using the magnetization-prepared rapid gradient echo sequence (MPRAGE; TR/TE = 2530/4.94 ms, slice number = 176). Structural MRI acquisition took 2 × 6:03 min. fMRI scans were obtained using a T2*-weighted echoplanar image (EPI) pulse sequence (TR/TE = 2000/30 ms, slice number = 32, voxel size = 3 × 3 × 3 mm^3^, slice gap = 0.75 mm). The functional imaging paradigm comprised four scanning blocks with a duration of 5:36 min, respectively.

### Experimental design and statistical analysis

#### Sensory-tactile stimulation paradigm

**Figure 1.**
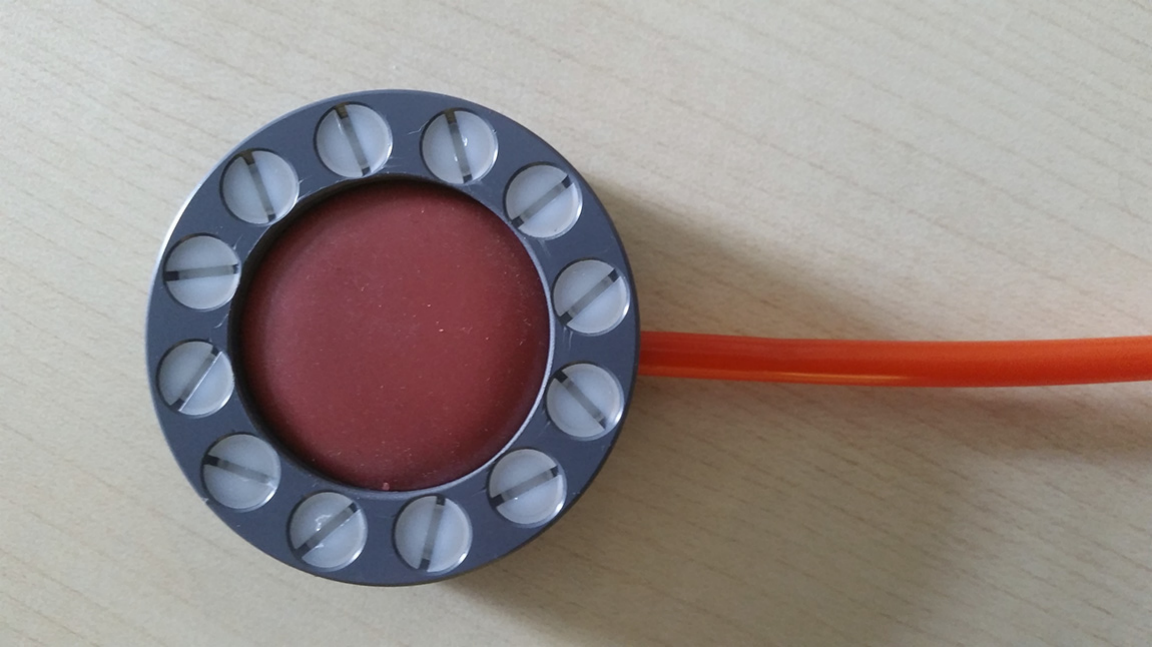
Device for sensory-tactile stimulation of the clitoral region and dorsum of the right hand. The stimulus is delivered via a noninvasive air-controlled oscillating membrane with a compression of ∼0.1 bar.

We developed an MRI-compatible sensory-tactile stimulation paradigm that allows for administering a defined focal sensory stimulus to the clitoral region (see Fig. 1). The stimulation was administered using a noninvasive air-controlled oscillating membrane with a compression of ∼0.1 bar. Women were asked to place the membrane below the mons pubis on the clitoral area above standardized disposable underwear. The sensory-tactile device was fixed with elastic tape and a flexible Velcro belt. Sensory-tactile stimulation of the dorsum of the right hand was used as a control condition, given that the S1 representations of the dermatomes of the genital region and the hand are well distinguishable (Roux et al., 2018).

The paradigm was performed in an ABBA versus BAAB block design with stimulation of either the clitoral region (A) or the dorsum of the right hand (B) interspersed with 10-s periods of no stimulation. Each of the four runs started with a period of no stimulation and included a total of eight clitoral and eight dorsum manus stimulation phases. The order of these phases was fixed and counterbalanced between women. Synchronization of the trigger pulses from the MRI scanner and the timing of the stimulation was controlled using Presentation (Neurobehavioral Systems Inc.). During the sensory-tactile stimulation, subjects were asked to fixate a cross on a screen. One woman completed only three runs.

Pleasantness and sexual arousal during clitoral stimulation were assessed after each run using a seven-point visual analog scale. Subjects were instructed to use a fiber-optic response box, indicating changes in pleasantness and sexual arousal. We then computed combined ratings on overall pleasantness and sexual arousal after the scan. We further inquired on the subjective appropriateness of the location of the clitoral membrane during the experiment as well as on sensations in other body parts during clitoral stimulation. There was no evidence for dislocation of the stimulation membrane in the sample.

#### Localization of genital field

Statistical parametric mapping 12 (SPM12; Wellcome Trust Center for Neuroimaging, University College London, London, United Kingdom) was used to perform functional image analysis to localize the genital field in S1. Standard spatial preprocessing of functional images, including realignment and coregistration to T1 image, was separately performed for each of the four scanning blocks. Data were high-pass filtered with a default cutoff period of 128 s to correct for slow drift artifacts. There was no head motion above 3.0 mm and 3.0° of maximal translation and rotation in any direction throughout a scanning block.

After standard spatial preprocessing, fMRI data were analyzed using a general linear model (GLM). The two within-subject conditions of interest (10 s of either clitoral or hand stimulation alternating with 10 s of rest) were modeled using a boxcar function convolved with a canonical hemodynamic response function (HRF). Activation maps were calculated with *t* tests for contrasts between the two regressors of the design matrix, resulting in individual patterns of neural activation in response to clitoral versus hand stimulation. We identified an activated region in SI for each participant at *p* < 0.001 without correction or *p* < 0.05 with family-wise error (FWE) correction for multiple comparisons. Individual neural activations were overlaid onto coregistered anatomic scans and saved as individual regions of interest (ROIs) for the left and right hemisphere, respectively. Individual ROI was multiplied with the t-score map corresponding to the individual contrast image to delineate the most activated vertices within the individually defined ROI. We purposely did not perform spatial normalization to a standard stereotaxic space (Montreal Neurologic Institute EPI template; MNI) or smoothing of the images to allow for subsequent cortical thickness analyses within the individually mapped ROI in native space of the anatomic images, as needed for the use-dependent plasticity analyses. To determine variability of the location of the genital field and hand representation in S1 between women, coordinates of peak neural activation were transformed in MNI space. Barycentre and dispersion across individually mapped fields were computed by averaging individual coordinates in MNI space.

To additionally localize the genital representation field in S1 on the group level, a random effects GLM was estimated across subjects. For this, individual contrast maps were spatially normalized to a standard MNI template and resampled to an isotropic spatial resolution of 3 × 3 × 3 mm^3^. Furthermore, data were spatially smoothed with a 6-mm full-width at half-maximum isotropic Gaussian kernel. Whole-brain group-level analysis with *t* tests contrasting neural response to the two with-subject factors genital stimulation versus stimulation of the dorsum of the right hand was thresholded at *p* < 0.05 with FWE-correction for multiple comparisons. Coordinates of the group-based neural activation reflecting the genital field are given in standard MNI space.

These statistical analyses and figures were computed using MATLAB (MathWorks, version 9.6.)

#### Anatomical image segmentation and surface-based morphometry (SBM; CAT12)

Automated image segmentation included (1) spatial registration (affine registration to tissue probability map); (2) initial SPM unified segmentation and skull stripping; (3) local intensity transformation to reduce tissue inhomogeneities (local adaptive segmentation; Dahnke et al., 2012); (4) volumetric segmentation of gray matter (GM), white matter (WM), and CSF, as well as GM-WM and GM-CSF, providing a more accurate segmentation (Tohka et al., 2004); (5) spatial normalization/DARTEL registration (Ashburner, 2007); (6) central surface estimation (projection-based thickness method; Dahnke et al., 2013); (7) topology correction (Yotter et al., 2011a); (8) surface inflation (spherical mapping; Yotter et al., 2011b) and spherical atlas registration (resampling; Yotter et al., 2011c), and default merging of hemispheres.

#### Thickness of individually mapped genital field

The Computational Anatomy Toolbox 12 (CAT12; Christian Gaser, Structural Brain Mapping Group, Jena University Hospital, Jena, Germany) for SPM12 was used to perform cortical SBM of the anatomic scans. Image segmentation was conducted using an automated standard procedure. The individually defined ROIs for the clitoris and the dorsum of the right hand were separately mapped onto individual native space cortical surfaces of the left and right hemisphere. After cortical surface registration, mean thickness of the 10 functionally most active vertices within the individually mapped ROIs was separately calculated for each hemisphere in each woman. Cortical thickness at each vertex was calculated as part of central surface estimation (Dahnke et al., 2013), describing the closest distance between the inner surface (WM/GM boundary) and the outer surface (GM/pial boundary) at each vertex of the tessellated brain surface (Fischl and Dale, 2000; Dahnke et al., 2013).

#### Use-associated structural variation of the genital field

We assessed mean frequency of sexual intercourse per week using a standardized biographic questionnaire to quantify sexual intercourse within the past 12 months and in five-year ranges since the onset of the first sexual genital contact. As noted above, we excluded sexual anxiety, discontent or dysfunction as well as dissociation during intercourse using established questionnaires (McGahuey et al., 2000; Berner et al., 2004; Brenk-Franz and Strauß, 2011; Hansen et al., 2012; Hoyer et al., 2015; Müller, 2016). To associate cortical thickness measures of the individually mapped genital field with data on sexual behavior, we correlated individual cortical thickness with the mean frequency of sexual intercourse per week within the past 12 months. We further correlated cortical thickness of the individually mapped genital field with the frequency of sexual intercourse estimated across a longer time period since the first onset of sexual contact. As we calculated one correlation per hemisphere, we did apply a Bonferroni-correction for multiple comparisons to the results (α_corr_ = 0.025). Using partial correlation analyses, we used age, years since onset of sexual contact, and whole-brain cortical thickness as covariates to control for effects of these variables on genital field cortical thickness. Furthermore, correlations and partial correlations between left-hemispheric cortical thickness of the representation field of the right hand and frequency of sexual intercourse for either time window were calculated to confirm for region-specificity of use-associated variation. These statistical analyses and figures were computed using R Project for Statistical Computing (R Core Team, version 4.0.2) and IBM SPSS Statistics (IBM, version 27).

### Data availability

The datasets generated during and/or analyzed during the current study are available from the corresponding author on reasonable request.

### Code accessibility

Custom MATLAB Code (version R2018b, MathWorks) for SPM12 and CAT 12 will be provided on request.

## Results

### Demographic and behavioral data

Demographic and behavioral data are presented in Table 1. Mean age of the sample was 23.10 years (SD = 4.35). The majority of women was of European descent, had a higher education, were heterosexual, lived in a monogamous partnership, and were right-handed. Seven women were on oral contraceptives. MR scans were distributed across menstrual cycle phase. Mean frequency of sexual intercourse in the past 12 months was reported to have been 1.91 times per week (SD = 1.30). Mean frequency of sexual intercourse since the onset of sexual contact was reported to have been 1.46 times per week (SD = 0.93). Importantly, behavioral data obtained during the sensory-tactile stimulation paradigm confirmed that the stimulation was not unpleasant and neither overly pleasant nor overly sexually arousing.

**Table 1.** Characteristics of the sample and behavioral data (*N* = 20)

Age, mean ± SD	23.10 ± 4.35
Ethnicity, *n* (%)	
European	18 (90%)
Middle East	1 (5%)
Asian	1 (5%)
Education, *n* (%)	
Enrolled in university	20 (100%)
Bachelor degree completed Master degree completed	6 (30%) 2 (10%)
Sexual orientation^1^, *n* (%)	
Heterosexual	17 (85%)
Bisexual	3 (15%)
Homosexual	0 (0%)
Partnership^1^, *n* (%)	
Monogamous partnership	14 (70%)
Polygamous partnership	1 (5%)
No partnership	5 (25%)
Sexual behavior^1^, mean ± SD	
Frequency of sexual intercourse/week since onset of sexual contact	1.46 ± 0.93
Frequency of sexual intercourse/week within the past 12 months	1.91 ± 1.30
Perceived pleasantness/sexual arousal during sensory-tactile clitoral stimulation^1,2^, mean ± SD	
Pleasantness	5.10 ± 0.91
Sexual arousal	4.00 ± 1.41
Contraception and menstrual cycle^1^, i (%)	
Hormonal contraception	7 (35%)
Follicular phase	5 (25%)
Ovulation	3 (15%)
Luteal phase	3 (15%)
Irregular menstrual cycle	2 (10%)
Handedness^1^, *n* (%)	
Right-handed	18 (90%)
Left-handed	2 (10%)

Values are mean ± SD or *n* (%).

^1^Information derived from self-report.

^2^Seven-point visual analog scale: 1 = unpleasant/no sexual arousal, 7 = overly pleasant/increased sexual arousal.

### Functional mapping of the female genital field: neural response to sensory-tactile stimulation

**Figure 2.**
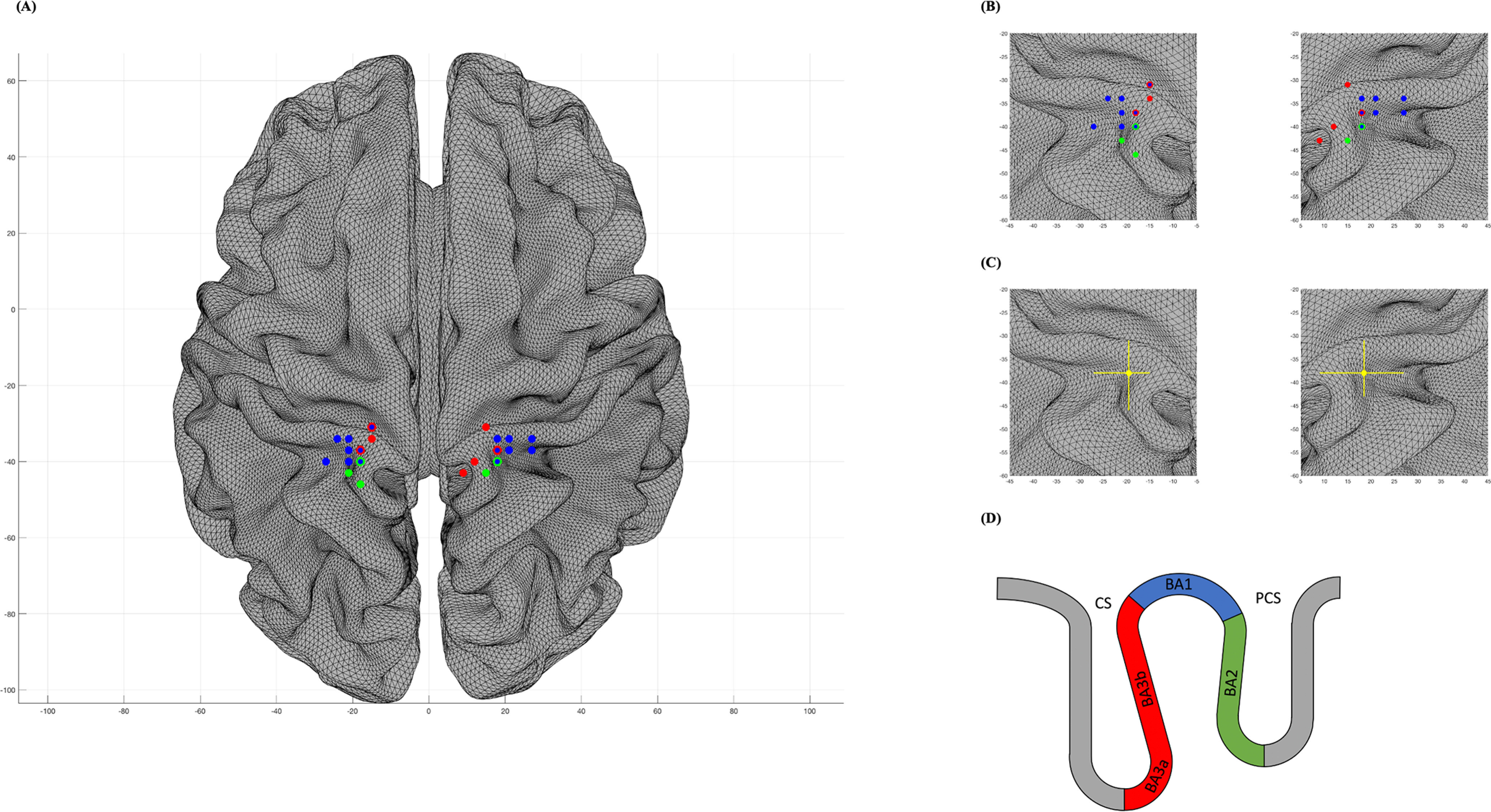
Interindividual variability of the genital somatosensory cortex in the MNI space. ***A***, Bilateral distribution of single subjects' representation of the clitoris in S1. Brodmann classification was based on probabilistic cytoarchitectonic maps (JuBrain Anatomy Toolbox v3.0; Simon Eickhoff, Institut für Neurowissenschaften und Medizin, Forschungszentrum Jülich, Jülich, Germany). Bicolored data points indicate overlapping Brodmann areas, depending on the *z*-coordinate in the transverse plane (see ***D***). ***B***, Detailed distribution over the two hemispheres, respectively. ***C***, Barycentres of the genital representations (shown in dots) on the left and right hemisphere with amplitude bars representing the dispersion (shown in lines). MNI barycentres of the genital representation on the left hemisphere [*x* = −19.5 (SE: ±2.8, range: −27 to −15), *y* = −38 (SE: ±3.6, range: −46 to −31), *z* = 72 (SE: ±4.3, range: 62–80)] and right hemisphere [*x* = 18.5 (SE: ±4.3, range: 9 to 27), *y* = −38 (SE: ±2.8, range: −43 to −31), *z* = 71.5 (SE: ±4.3, range: 62–80)]. ***D***, Schematic representation of the anterior parietal areas BA3a, BA3b, BA1, and BA2, indicating that all data points lay within the postcentral gyrus based on a probabilistic atlas of human cortical brain areas (Harvard–Oxford macroanatomical atlas).

Sensory-tactile stimulation of the clitoral region (relative to right hand) induced significant focal neural activations in S1. Sixteen women exhibited bilateral neural activations in S1. For four women, a significant activation was found in either the right or the left hemisphere only. Table 2 delineates individual MNI coordinates with the respective p value thresholds and t scores of the sensory foci for clitoral stimulation. Individual focal neural activations occurred in Brodmann areas 1, 2, and 3a/3b (BA1–BA3) of the postcentral gyrus for all women. Within BA1–BA3, there was distinctive individual variability of the precise location of the neural activation in response to stimulation of the clitoral region. Figure 2 shows the individual localization of the clitoral somatosensory representation in normalized stereotaxic coordinates (MNI space).

**Table 2.** Individual and group cortical activations in response to sensory-tactile stimulation of clitoris or dorsum of the right hand

Single subject	Genital representation Left hemisphere Center of gravity (*x*, *y*, *z*)	*t* value	*p* threshold	Cortical thickness	Genital representation Right hemisphere Center of gravity (*x*, *y*, *z*)	*t* value	*p* threshold	Cortical Thickness	Hand Representation Left hemisphere Center of gravity (*x*, *y*, *z*)	*t* value	*p* threshold	Cortical thickness
1	−21, −40, 74	14.48	FWE 0.05	2.3309	18, −40, 80	5.75	FWE 0.05	2.5585	−42, −37, 59	4.92	FWE 0.05	2.9968
2	−24, −34, 77	3.35	Uncorr. 0.001	2.0890	15, −31, 71	2.26	Uncorr. 0.001	1.5174	−33, −31, 68	1.68	Uncorr. 0.001	2.4194
3	---	---	---	---	15, −43, 62	3.22	Uncorr. 0.001	2.1214	−27, −31, 68	4.56	FWE 0.05	2.4300
4	−18, −40, 62	10.12	FWE 0.05	2.2791	---	---	---	---	−39, −34, 65	4.50	FWE 0.05	2.3970
5	−18, −46, 68	4.83	Uncorr. 0.001	2.1010	---	---	---	---	−39, −40, 62	2.70	Uncorr. 0.001	2.8083
6	−21, −40, 71	3.01	Uncorr. 0.001	2.5363	18, −37, 71	6.00	Uncorr. 0.001	2.1836	−36, −28, 65	4.26	FWE 0.05	1.7383
7	−21, −34, 80	9.01	FWE 0.05	2.2215	27, −34, 71	17.29	FWE 0.05	1.7881	−45, −31, 62	13.59	FWE 0.05	2.2301
8	−21, −40, 71	14.13	FWE 0.05	2.4748	21, −37, 71	10.29	FWE 0.05	2.7262	−39, −22, 65	3.81	FWE 0.05	2.1545
9	−15, −34, 71	4.62	Uncorr. 0.001	2.3893	18, −37, 65	6.31	Uncorr. 0.001	2.0032	---	---	---	---
10	−15, −31, 65	7.06	Uncorr. 0.001	2.4279	18, −40, 74	7.68	Uncorr. 0.001	2.6370	−36, −28, 65	2.68	FWE 0.05	2.1161
11	−18, −40, 68	11.76	FWE 0.05	2.6785	18, −34, 74	10.04	FWE 0.05	2.0501	−36, −25, 65	8.55	FWE 0.05	2.3915
12	−21, −37, 77	7.68	Uncorr. 0.001	2.4222	21, −37, 68	12.56	Uncorr. 0.001	2.1976	−42, −37, 56	7.05	FWE 0.05	2.3930
13	---	---	---	---	27, −37, 71	3.34	Uncorr. 0.001	2.2787	−42, −40, 56	5.38	Uncorr. 0.001	2.7626
14	−15, −31, 77	4.52	Uncorr. 0.001	1.7867	12, −40, 71	10.24	Uncorr. 0.001	2.0809	−33, −28, 62	4.93	Uncorr. 0.001	1.5778
15	−18, −37, 68	6.99	FWE 0.05	2.2393	18, −40, 80	8.92	FWE 0.05	2.3916	−45, −31, 56	2.57	Uncorr. 0.001	2.8190
16	−21, −40, 74	6.46	FWE 0.05	2.0462	21, −34, 77	9.89	FWE 0.05	2.2819	−33, −34, 53	2.31	Uncorr. 0.001	1.9530
17	−21, −43, 71	3.84	Uncorr. 0.001	2.8488	09, −43, 68	4.66	Uncorr. 0.001	2.3182	−45, −28, 62	3.21	FWE 0.05	2.8617
18	−18, −37, 74	6.46	FWE 0.05	2.4128	18, −37, 71	7.80	FWE 0.05	2.5398	−36, −28, 59	3.06	Uncorr. 0.001	1.6492
19	−18, −37, 74	7.48	FWE 0.05	1.8379	18, −40, 71	17.96	FWE 0.05	2.0445	−39, −37, 62	7.24	FWE 0.05	2.1116
20	−27, −40, 71	2.83	Uncorr. 0.001	2.6396	18, −40, 71	5.80	Uncorr. 0.001	2.3120	−36, −28, 65	2.25	Uncorr. 0.001	1.7425
Group	Center of gravity (*x*, *y*, *z*)	*t* value	*p* threshold		Center of gravity (*x*, *y*, *z*)	*t* value	*p* threshold		Center of gravity (*x*, *y*, *z*)	*t* value	*p* threshold	
	−18, −34, 72	7.72	FWE 0.05		18, −40, 68	10.26	FWE 0.05		−33, −31, 62	6.13	Uncorr. 0.001	

Coordinates indicate the somatosensory localizations in the *x* (mediolateral, with positive values for right hemisphere and negative values for left hemisphere), *y* (rostrocaudal, with negative values for caudal), and *z* (dorsoventral, with positive values for dorsal) axes in the MNI space. Individual and group activations were significant at *p* < 0.001 without correction or *p* < 0.05 with FWE correction for multiple comparisons. –, no functional activations detected.

**Figure 3.**
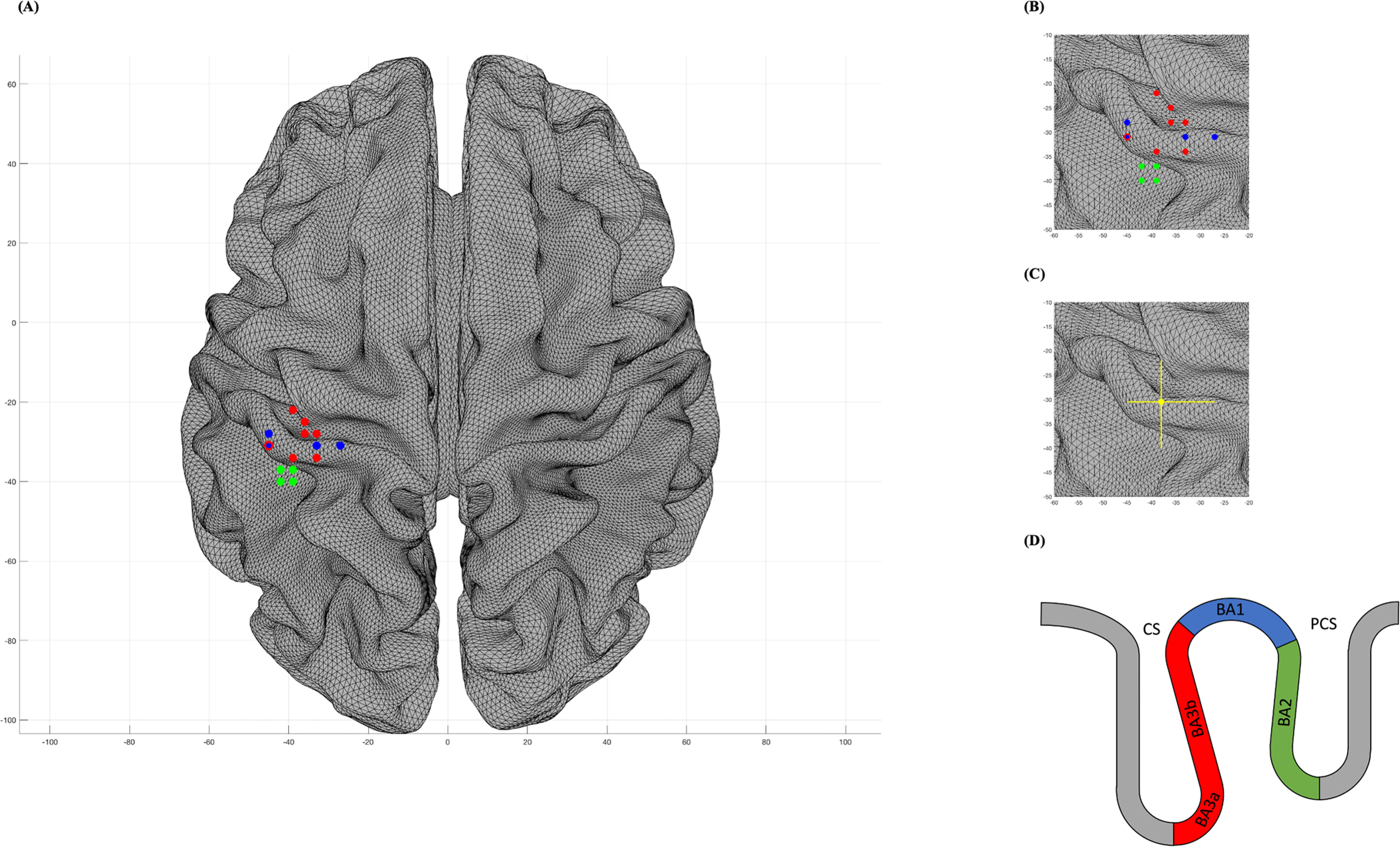
Interindividual variability of the hand somatosensory representation in the MNI space. ***A***, Contralateral distribution of single subjects' representation of the right dorsum of the hand in S1. Brodmann classification was based on probabilistic cytoarchitectonic maps (JuBrain Anatomy Toolbox v3.0; Simon Eickhoff, Institut für Neurowissenschaften und Medizin, Forschungszentrum Jülich, Jülich, Germany). Bicolored data points indicate overlapping Brodmann areas, depending on the *z*-coordinate in the transverse plane (see ***D***). ***B***, Detailed distribution over the left hemisphere. ***C***, Barycentre of the hand representation (shown in dots) on the left hemisphere with amplitude bars representing the dispersion (shown in lines). MNI barycentres of the hand representation on the left hemisphere [*x* = −38 (SE: ±4.3, range: −45 to −27), *y* = −30.5 (SE: ±4.3, range: −40 to −22), *z* = 62 (SE: ±5.0, range: 53–74)]. ***D***, Schematic representation of the anterior parietal areas BA3a, BA3b, BA1, and BA2, indicating that all data points lay within the postcentral gyrus based on a probabilistic atlas of human cortical brain areas (Harvard–Oxford macroanatomical atlas).

We next mapped the individual representation of the dorsum of the right hand for use in subsequent cortical thickness analyses. Sensory-tactile stimulation of the dorsum of the right hand (relative to clitoral region) induced significant contralateral focal neural activations in S1. Table 2 delineates individual MNI coordinates with the respective p value thresholds and t scores of the sensory foci for the stimulation of the right hand. Individual focal neural activations occurred in BA1–BA3 of the postcentral gyrus, with individual variability of the precise location of the neural activation. Figure 3 shows the individual localization of the somatosensory representation of the hand in normalized stereotaxic coordinates (MNI space) for the left hemisphere. There was no significant effect of handedness on functional activation of the hand representation. Of note, the location of the representation field of the clitoris and the representation field of the hand was somatotopically-ordered for each woman and commensurate with anatomic location.

**Figure 4.**
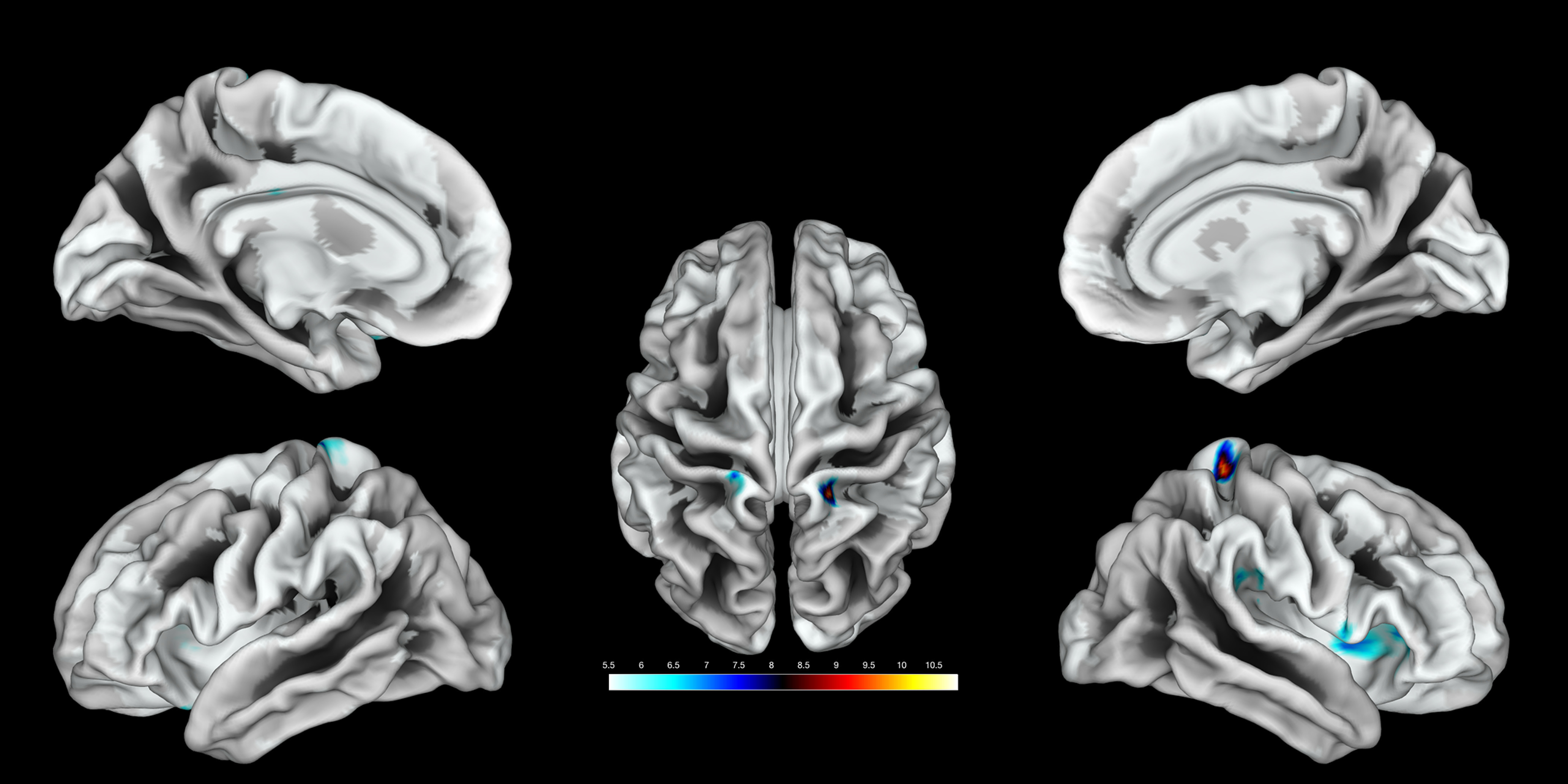
Cortical surface mapping of functional somatosensory activations of the random effects GLMs of sensory-tactile stimulation of the clitoral region (left hemisphere: *x* = −18, *y* = −34, *z* = 74; *T* = 7.72, *p*_FWE-corr_ = 0.024; right hemisphere: *x* = 18, *y* = −40, *z* = 68; *T* = 10.26, *p*_FWE-corr_ < 0.0001).

When analyzed at the group level across all women, GLMs revealed significant symmetric dorsolateral neural activations in S1 in response to stimulation of the clitoris (relative to hand) in both hemispheres (left hemisphere: *x* = −18, *y* = −34, *z* = 74; *T* = 7.72, *p*_FWE-corr_ = 0.024; right hemisphere: *x* = 18, *y* = −40, *z* = 68; *T* = 10.26, *p*_FWE-corr_ < 0.0001). Of note, no other significant neural activations were observed at the group level in response to the stimulation of the clitoral region, suggesting that the stimulation paradigm specifically targeted the genital field and was not overly arousing. Figure 4 shows normalized stereotaxic coordinates (MNI space) for the group location mapped onto the cortical surface.

### Use-associated structural variation of the female genital field: SBM

**Figure 5.**
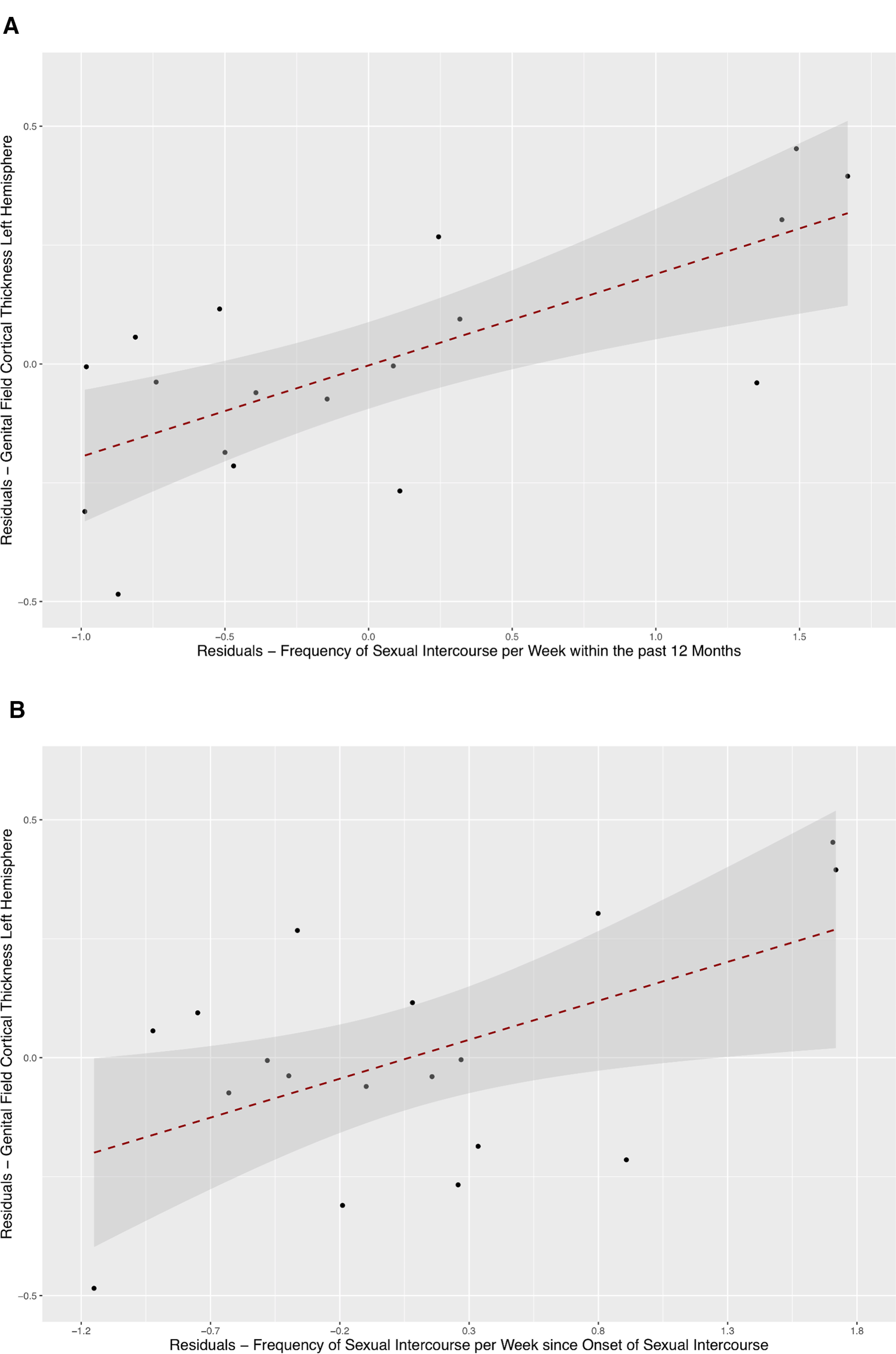
***A***, Scatter plot with SE on the correlation between frequency of sexual intercourse per week within the past 12 months and left-hemispheric genital field cortical thickness. Data points are plotted as residuals with correction for covariates. ***B***, Scatter plot with SE on the correlation between frequency of sexual intercourse per week since onset of sexual contact and left-hemispheric genital field cortical thickness. Data points are plotted as residuals with correction for covariates. (Partial correlation values of covariates with genital field cortical thickness: age: *r* = –0.460, *p* = 0.055; years of sexual intercourse: *r* = –0.380, *p* = 0.120; whole-brain cortical thickness: *r* = 0.309, *p* = 0.213.)

We mapped individual ROIs for the genital field (representing the 10 most activated vertices per hemisphere during clitoral stimulation) onto native cortical surfaces for each subject and estimated cortical thickness of the individual genital representation field (for individual data, see Table 2). Partial correlation analysis controlling for age, years since onset of sexual contract, and whole-brain cortical thickness revealed a significant positive correlation between cortical thickness of the individually-mapped left-hemispheric genital field and the frequency of sexual intercourse within the past 12 months (*r* = 0.701, *p* = 0.004; corrected *p* < 0.05). Similarly, longer-term frequency of sexual intercourse estimated since the onset of sexual contact was significantly correlated with thickness of the individually-mapped left-hemispheric genital field in a partial correlation analysis (*r* = 0.538, *p* = 0.039). Partial correlation analyses between cortical thickness of the right-hemispheric genital field and frequency of sexual intercourse did not reveal any significant effects, suggesting lateralized use-associated structural variation. Figure 5 shows scatterplots of left genital field thickness against frequency of sexual intercourse for the past 12 months and frequency of sexual intercourse since the onset of sexual contact, plotted as residuals corrected for covariates. Of note, menstrual cycle phase was not significantly associated with thickness of the genital field.

To confirm the specificity of this effect, we mapped individual ROIs for the representation of the hand (representing the 10 most activated vertices in the left hemisphere in response to stimulation of the right hand) onto native cortical surfaces for each woman and estimated cortical thickness of the individual representation field of the hand (for individual data, see Table 2). Importantly, cortical thickness of the hand representation was not significantly associated with frequency of sexual intercourse at either time window, with or without correction for the effects of covariates, reflecting a highly specific use-dependent effect for the sensory field involved in the specific behavior.

## Discussion

We present novel evidence on the precise location of the female genital representation field and its capacity for use-associated structural variation. Using functional mapping during sensory-tactile stimulation of the clitoral region, we show focal bilateral neural activations within the dorsolateral postcentral gyrus in S1. We show that the individual location of peak neural activations in response to clitoral stimulation varies considerably between women. We applied cortical surface analysis to the individually-mapped ROI to compute structural thickness of the genital field. Correlating the individually-mapped morphologic data with behavioral data on sexual contact, we provide first evidence that thickness of the genital field varies as a function of frequency of genital intercourse in the past 12 months and lifetime, in line with use-associated plasticity.

Our results are noteworthy in several ways. To localize the female genital field, we measured neural response in a tactile-sensory stimulation paradigm that delivers a physiologically valid stimulus as opposed to a previous study using electrical stimulation of the clitoris (Michels et al., 2010). Furthermore, our tactile-sensory stimulation paradigm did not involve touching of body parts adjacent to the clitoris nor did it induce marked sexual arousal as opposed to previous studies using self-delivered or partner-delivered stimulation (Georgiadis et al., 2006, 2009, 2010; Komisaruk et al., 2011). The sole other study that used a sensory-tactile nonarousing stimulation paradigm to localize the genital field was limited to males (Kell et al., 2005). Our stimulation paradigm induced focal targeted neural activations, without inducing neural activation in other brain regions, at comparatively (Kell et al., 2005; Michels et al., 2010) high levels of statistical significance without using somatosensory template masks. Therefore, our data provide unequivocal information about the location of the female genital field and represent a significant methodological advance compared with previous studies that yielded conflicting results (Georgiadis et al., 2006, 2009; Michels et al., 2010; Komisaruk et al., 2011), likely because of confounding factors inherent to stimulation paradigms used in these studies (Pratt et al., 1980; Forss et al., 1994). On a group level, the mean location of the female genital field in the dorsolateral postcentral gyrus, identified in our study, corresponds with the location reported in two of the previous studies in females using electrical (Michels et al., 2010) or partner-delivered manual stimulation (Georgiadis et al., 2006) as well as with the location reported for males in the above-referenced study using sensory-tactile stimulation in males (Kell et al., 2005). Our results confirm a somatotopically-ordered representation of the female clitoris, adjacent to the representation of the hips and upper legs and commensurate with anatomic location, and disprove displaced location in the mesial wall of the precentral lobe. Our results provide independent confirmation for the revision (Kell et al., 2005) of the original homunculus (Penfield and Rasmussen, 1950) and extend the validity of the revised homunculus to women. Our results confirm a bilateral somatosensory representation of the anatomically centered clitoris, in line with histologic mapping data on the localization and bilateral representation of the rat genital cortex (Lenschow et al., 2016; Lauer et al., 2017; Lenschow and Brecht, 2018).

Our results suggest profound variability of the individual location of the genital field within the dorsolateral part of S1 with individual peak activations clearly deviating from the group mean. This means that any study looking at structural variation of the genital field as a function of certain conditions, such as sexual behavior, sexual abuse or sexual dysfunction, must necessarily implement individual mapping of the genital field and compute data, i.e., cortical thickness, on an individual level. Clearly, only by using individually-mapped ROIs, such studies yield precise reliable surface-based parameters for association with specific conditions.

We computed data on structural thickness of the genital field in individually-mapped ROIs, based on the 10 most activated vertices per hemisphere for each woman. We show that individual thickness of the left genital field associates with frequency of sexual intercourse. The association was stronger for genital intercourse within the past 12 months. While less pronounced, the association was significant for lifetime genital contact. Frequency of genital intercourse was not associated with thickness of the representation field of the right hand nor with thickness of the entire cortical mantle, confirming a specific association between genital touch and genital field thickness. This is compatible with the idea that the female genital field has capacity for structural plasticity depending on its use, commensurate with the general “use-it-or-lose-it” principle of experience-dependent plasticity (Hebb, 1947; Elbert and Rockstroh, 2004; Draganski and May, 2008). While injury-dependent or use-dependent plasticity in the human somatosensory cortex has been reported (Elbert et al., 1994, 1995; Flor et al., 1995; Foell et al., 2014), our results are the first to document structural variation of genital field thickness associated with more or less frequent normative use. Our results are in line with findings from animal studies showing that genital brushing during puberty resulted in lateral expansion of the rat and mouse genital cortex (Lenschow et al., 2017; Sigl-Glöckner et al., 2019). Cortical plasticity serves to enhance the efficiency of processing of behaviorally-relevant inputs and represents an adaptive response (Trachtenberg et al., 2002; Markham and Greenough, 2004; Feldman and Brecht, 2005; May, 2011). In an earlier study, we observed decreased thickness of the genital cortex after exposure to childhood sexual abuse, suggesting that highly aversive and developmentally inappropriate sexual stimulation may limit somatosensory representation to decrease processing of detrimental input (Heim et al., 2013).

Several mechanisms might contribute to dynamic use-associated structural plasticity of the genital field. Structural thickening of the mature cortex as a function of use most likely reflects formation of new synapses by axonal sprouting, dendritic arborization, and dendritic spine growth rather than induction of new neurons through neurogenesis (Markham and Greenough, 2004; Feldman and Brecht, 2005; Feldman, 2009; May, 2011). There is substantial evidence on the central role of glutamatergic synapses in mediating plasticity, reflecting rapid components of NMDA receptor-dependent long-term potentiation (LTP) and long-term depression (LTD; Buonomano and Merzenich, 1998; Feldman, 2009). Another mechanism contributing to use-associated structural plasticity may involve alterations in glial-cell mediated myelination (Timmler and Simons, 2019). While oligodendrogenesis is rare (Yeung et al., 2019), the presence of large numbers of premyelinating oligodendrocytes in the human cortex may enable adaptive myelination to adapt conduction velocity to functional demand (Gibson et al., 2014). Future studies in humans should use novel imaging tools that allow for assessing cortical myelin density (Amunts and Zilles, 2015) to study genital field plasticity. Further, neural activation in response to somatosensory stimulation depends on axonal input from the thalamus (Feldman, 2009). When removing afferent somatosensory input from the thalamus, dendritic spine numbers of somatosensory cortical neurons attenuate (Lendvai et al., 2000). When exposing rats to genital touch or sexual contact during puberty, invading thalamo-cortical afferents promote the expansion of the female genital cortex (Lenschow et al., 2016). Future studies on genital field plasticity should therefore include assessments of thalamo-cortical connectivity and myelination.

It must be noted that use-associated variation of structural thickness of the female genital field in our study was limited to the left hemisphere. This lateralized effect is puzzling given that the neural representation of the clitoris is bilateral. Left-hemispheric dominance of neural plasticity has been reported for learning-dependent structural change after coordination and motor skill training (Draganski et al., 2004; Taubert et al., 2010; Rogge et al., 2018). Such lateralized plasticity may reflect hemispheric specialization (Serrien et al., 2006). In the above referenced study (Heim et al., 2013), thinning of the genital field after sexual abuse was limited to the left hemisphere. While we cannot comprehensively explain these findings, one plausible mechanism may involve lateralized limbic-cortical modulation of sensory afferent inputs into the genital field, leading to unilateral associations of sexual behavior with genital field morphology.

While our localization of the female genital field was experimental in nature, our investigation of the capacity of the genital field for structural variation as a function of genital contact was cross-sectional and relied on retrospective self-report of genital intercourse. Our results align with the general principle of an association between frequency of genital intercourse and structural variation, albeit the direction of effect is a matter of discussion. It is conceivable that thickness of the genital field may drive frequency of sexual intercourse. Results from animal models provide causal that clitoral stimulation drives genital field thickness (Lenschow et al., 2016; Lenschow and Brecht, 2018). Future prospective studies or studies exploiting quasi-experimental conditions, such as induction of behavior change during sexual therapy, are needed to establish causality.

In conclusion, we provide an unequivocal localization of the female genital field in S1 and support for use-associated plasticity of the human genital field. On a secondary level, our findings support the notion that studies investigating change of the human genital field must map the field individually. Our results pave the way for future research into the plasticity of the human genital field as a function of normal or adverse experience as well as genital field structure, function and plasticity in pathologic conditions, such sexual dysfunction, sexual deviation, or sexual risk-taking behavior.
